# Rapid Determination of Molybdenum (VI) in Water Using Phenylfluorone-Modified Test Strips Combined with Colorimetry and LAB Color Space Analysis

**DOI:** 10.3390/s26030885

**Published:** 2026-01-29

**Authors:** Xingping Li, Daiwei Zhuang, Xiaoling Liu, Hongbing Luo, Ke Zhang, Bing Jiang, Wei Chen, Wancen Xie

**Affiliations:** 1 School of Ecological Engineering, Henan Forestry Vocational College, Luoyang 471002, China; 2 Department of Municipal Engineering, School of Civil Engineering, Sichuan Agricultural University, Chengdu 611830, China; 3 School of Environment, Nanjing University, Nanjing 210023, China; 4 Department of Information Engineering, Sichuan Water Conservancy Vocational College, Chengdu 611231, China; 5 College of Environmental Sciences, Sichuan Agricultural University, Chengdu 611130, China; 6 Sichuan Higher Education Engineering Research Center for Disaster Prevention and Mitigation of Village Construction, Sichuan Agricultural University, Chengdu 611830, China; 7 Business and Tourism School, Sichuan Agricultural University, Chengdu 611830, China

**Keywords:** test strips, molybdenum (VI), phenylfluorone, portable devices, quick detection

## Abstract

Excessive molybdenum (VI) (Mo (VI)) in water threatens environmental safety and human health, yet rapid on-site methods for Mo (VI) determination remain limited. Here, we propose a rapid method for Mo (VI) determination using phenylfluorone (PF)-modified test strips with dual readouts: visual colorimetry and image-based analysis in the CIELAB (Lab*) color space, and demonstrate its applicability using urban park water samples. Based on visual colorimetry, a standard color card was established, providing a screening range of 0.08 to 0.8 mg L^−1^ (A blank (0 mg L^−1^) was used as the baseline reference). Moreover, by the LAB color space, the linear relationship between the color development results of the PF-modified test strip and the A channel conforms to y = 21.08 + 8.82x (R^2^ = 0.992), with a detection range of 0–0.8 mg L^−1^. The total detection time was reduced to 2.5 min. To evaluate accuracy in real matrices, influent, midstream, and effluent samples from Chengdu Living Water Park were analyzed, with UV-vis spectrophotometry used as the reference method. The test-strip results agreed well with UV-vis spectrophotometry, with relative errors below 5%. Overall, this study provides a portable, rapid, and accurate method for the detection of Mo (VI) in water, and has potential application prospects in the field of water environment detection in the future.

## 1. Introduction

With rock weathering and the large-scale application of molybdenum minerals in industrial activities, the circulating concentration of Mo in human life and aquatic environments is gradually increased [1]. The maximum concentration limit of Mo in both the “Environmental Quality Standards for Surface Water of China” [2] and the “Standards for Drinking Water Quality of China” [3] is 0.07 mg L^−1^. However, mining activities, tailings leakage, and ore-bearing wastewater discharge can markedly elevate Mo levels in surrounding surface water and groundwater, and such contamination has been reported in multiple mining-impacted regions worldwide [4,5,6,7,8,9,10,11,12]. Once released, excessive Mo may spread through rivers and aquifers, posing potential risks to water safety and drinking-water security [13]. Although molybdenum is an essential trace element (daily intake: 0.15–0.5 mg) [14], excessive exposure has been associated with adverse health outcomes such as gout as well as possible kidney and cardiovascular damage [15]. For example, residents in two Armenian villages showed a markedly increased incidence of gout after consuming crops irrigated with Mo-contaminated water sources [16]. Moreover, Mo-enriched water may further flow into cities through rivers and groundwater [17], and urban parks often contain water bodies that can come into direct contact with visitors. Therefore, rapid and effective detection of Mo in urban park water environments is of great significance to environmental safety and human health.

In natural environments, Mo mainly exists as Mo (IV) and Mo (VI), and Mo (VI) is generally the predominant and more mobile species in oxic waters [18]. Mo (IV) is typically associated with reducing/anoxic environments (e.g., sediments and porewaters), where it tends to occur as sparingly soluble reduced phases and is therefore much less mobile in the aqueous phase. In contrast, Mo (VI) is predominantly present as soluble molybdate under oxic conditions and thus represents the main form encountered in natural waters, which is also the target species of concern in this study [19]. At present, the instruments used to detect Mo (VI) in water include ICP-AES, ICP-MS, GF-AAS, UV-vis spectrophotometer, ion-exchange resin, and X-ray spectrometry, which are professional techniques with high precision. However, these methods also have disadvantages such as a large footprint, poor portability, complex operation, and high analytical cost [20]. Therefore, there is an urgent need to develop a portable, simple, and low-cost method for the rapid detection of Mo (VI).

Test strips have gradually become a research hotspot for rapid detection instruments in environmental and medical applications [21] in recent years because of their portability, low cost, simple operation, single-use format, and rapid response [21]. Wang et al. used the test strip modified with anion-inserted layered double hydroxide to quickly and simply detect heavy metal ions [22]. Based on a fluorescence affinity immunoassay, glycated hemoglobin can be rapidly measured [23]. Lv et al. designed a multifunctional diaryl fluorescent test paper containing a recognition unit to detect Cd^2+^ and Zn^2+^ in actual water samples [24]. Based on a molecularly imprinted sensitive membrane, a new test strip was reported for point-of-care detection of TDG [25]. Test strips can be continuously innovated mainly because they can be combined with a variety of detection principles, demonstrating their strong adaptability [26]. Therefore, by using a test strip as a detection platform and combining it with the chemical detection method for Mo (VI), a rapid tool specifically designed for Mo (VI) can be developed. As a common method for Mo (VI) detection, colorimetry can quantify the concentration of the target analyte based on the color change before and after chemical reaction. Therefore, a rapid method for quantifying Mo (VI) concentration in water can be achieved by combining colorimetry with test strips.

**Figure 1:**
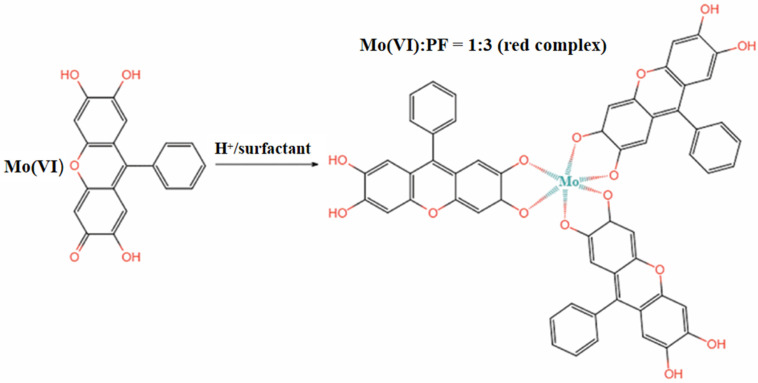
The reaction principle of PF and Mo (VI).

The reported colorimetric methods for Mo (VI) include the thiocyanate method [27], the hydrogen peroxide method [28], and the PF method [29]. Thiocyanate colorimetry is the most widely used method, but it suffers from unstable color development [30]. PF has been widely used in analytical chemistry in recent years because of its planar and conjugated π-bond structure, as well as its high sensitivity and selectivity [31]. In an acidic environment, PF binds to Mo (VI) under the solubilization and sensitization effects of surfactant. One Mo (VI) ion can bind with three PF molecules to form a red complex with a molar ratio of 1:3 [32] (see Figure 1). However, test strips cannot accurately quantify the relationship between color intensity and Mo (VI) concentration based only on naked-eye colorimetry; therefore, the color-development process should be converted into quantitative data with the help of other methods.

The LAB color space in Photoshop software is a color mode published by the International Commission on Illumination (CIE) in 1976 [33], which can include all colors seen by the human eye. L represents the brightness signal of the image. The A channel represents the red/green component, and the B channel represents the yellow/blue component. The combination of the A and B channels can express the colors seen by humans [34]. In the LAB color space mode in Photoshop software, each channel can be selectively opened or closed. When the L channel is turned off, it indicates that the interference from the external brightness signal during image acquisition is removed. When the A channel is viewed, it shows the color signal from red to green in the image. When the B channel is viewed, it indicates the color signal from yellow to blue in the image. When the A/B channels are opened, the selected image area has specific A/B values, which represent the color intensity of the image in the lab space [35]. In recent years, the LAB color space has been widely used in image processing because it can make up for the limitations of the RGB and CMYK color modes [36]. Wang et al. [37] quantified the relationship between gingival color and tooth health in the LAB color space. Jeong et al. [38] designed a robust lip-detection algorithm based on the A and B channels in the LAB color space and optimized the representation of lip color in images. Nelis et al. [39] quantitatively measured the color changes of pH strips, filter paper loaded with nanoparticles, and colored solutions using the LAB color space. Zhang et al. extracted the heart-rate pulse signal using the difference between channel A and channel B while reducing the interference of the brightness signal in the LAB color space [40]. Previous studies indicate that the LAB color space can provide more accurate and realistic color quantification because imagecolor can be digitized in this space [41]. Therefore, the LAB color space can be used to determine the color change of the test strip before and after reaction with Mo (VI) in water by establishing a mathematical model linking image color intensity (A/B values) to Mo (VI) concentration.

In this study, we developed a PF-immobilized paper test strip coupled with a dual-readout strategy (visual colorimetry and CIELAB-based image quantification) for rapid determination of Mo (VI) in water. The objectives were to optimize key operational parameters (surfactant type/composition, soaking time, solution pH, and color-development time), establish a practical calibration model and a standard color card for on-site use, and validate the method using influent, midstream, and effluent water samples collected from an urban park water system. The proposed approach provides a portable and low-cost tool with a rapid response (≈2.5 min), a visual screening range of 0.08–0.8 mg L^−1^, and satisfactory agreement with the reference UV-vis method in real-water matrices, demonstrating its potential for field applications.

## 2. Experimental

### 2.1. Materials

All chemicals were of analytical grade AR unless otherwise stated. Phenylfluorone (PF, AR), MoO_3_ (AR), TritonX-100 (laboratory grade), C_16_H_33_(CH_3_)_3_NBr(CTMAB) (AR), HCl (AR), NaOH (AR) were used for pH adjustment from Chengdu ShiJiFangZhou Ltd., Chengdu, China. Salts used for interference tests were all AR grade, including KNO_3_, NaCl, CaCO_3_, CuSO_4_, ZnSO_4_, MnSO_4_, FeCl_3_·6H_2_O, AlCl_3_, MgSO_4_, Na_2_C_2_O_4_, K_2_Cr_2_O_7_, NiCl, and CdCl_2_. Deionized water was used throughout.

### 2.2. Synthesis of PF-Modified Test Strips

In order to synthesize the PF-modified test strips, the whatman1 filter paper was first cut into 10 mm × 10 mm square pieces and then washed tree times with deionized water. Secondly, 5 mL of sulfuric acid with a concentration of 3 mol L^−1^ was used to dissolve 0.15 g of PF, which was then dissolved in 500 mL of absolute ethanol. Whatman1 filter papers were soaked in PF solution for 5 h and then heated to dryness on a hot plate at 60 °C. The dried PF-modified test strips were sealed and stored for subsequent experiments.

### 2.3. Experimental Detection Method

**Figure 2:**
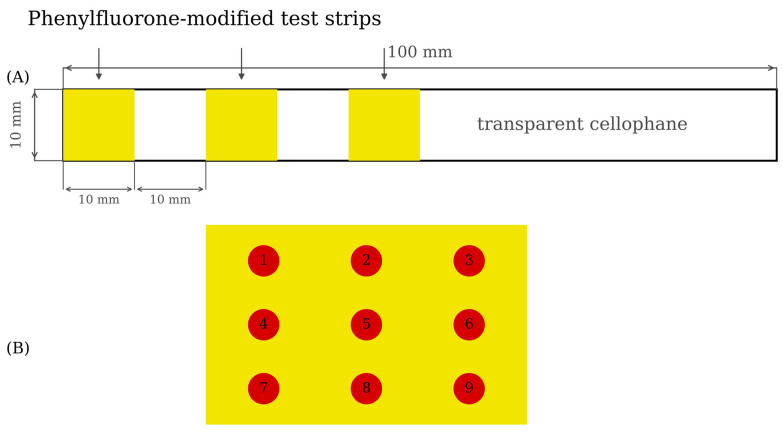
Schematic diagram of Mo (VI) test strip and 3 × 3 collection points of A value from Photoshop (**A**): Schematic diagram of Mo (VI) test strip (**B**): 3 × 3 color collection points of A value.

Three prepared PF-modified test strips were glued on one end of transparent cellophane (length was 140 mm, width was 10 mm) by double-sided adhesive to make Mo (VI) test strips (see Figure 2A).

The Mo (VI) test strip was placed in an acidic Mo (VI) solution for 1 min. then removed, and the excess solution was shaken off. The Mo (VI) test strip, after a period of color development, was placed in a self-made camera for photographing. The captured image was imported into the LAB color space in Photoshop, and then the L and B channels were turned off simultaneously. The A-channel value of the color area on the PF test strip was recorded with only the A channel turned on. The calculation method of the A-channel value is shown in Formula (1) [42].

(1) $$A=500\left[{\left(\frac{x}{x_{0}}\right)}^{\frac{1}{3}}-{\left(\frac{y}{y_{0}}\right)}^{\frac{1}{3}}\right]$$

where *x* and *y* are the tristimulus values of the color sample, and *x*_0_ and *y*_0_ are the tristimulus values of the standard illumination sample.

In order to ensure that the A channel could represent the color development result of a PF test strip, the average value of the A channel of the 3 × 3 color collection points on a PF test strip (see Figure 2B) was used as *A_average value_*. The calculation method of *A_average value_* was shown in Formula (2) [40].

(2) $$A_{average\;value}=(\frac{A_{1}+A_{2}+A_{3}\cdots A_{9}}{9})$$

where *A_n_* represents the value of the color collection point of *n*, and *n* is 1–9.

There were three PF test strips on a Mo (VI) test strip as parallel samples of the test results. The color development result of a Mo (VI) test strip was written as *S_A_*, which was equal to the average of the color development results of three PF test strips minus the color development result of the blank Mo (VI) test strip. The calculation method of *S_A_* was shown in Formula (3) [40].

(3) $$S_{A}=\left(\frac{A_{average\;value1}+A_{average\;value2}+A_{average\;value3}}{3}\right)-S_{A_{\mathrm{blank}}}$$

### 2.4. Characteristics of PF-Modified Test Strip

#### 2.4.1. Type of Surfactant

Three mixed solutions with a total volume of 20 mL and a volume ratio of 1:1 were prepared: a PF–deionized water mixed solution, a PF–Triton X-100 mixed solution, and a PF–CTMAB mixed solution. Whatman1 filter paper (10 mm × 10 mm) was washed three times with deionized water, and then soaked in three mixed solutions for 2 h. Then, the PF-modified test strips were dried and made into Mo (VI) test strips. The Mo (VI) test strips were immersed in 25 mL of 0.6 mg L^−1^ Mo (VI)-containing solution and allowed to stand for 1 min. After that, the test strips were taken out, and S_A_ was calculated.

#### 2.4.2. Volume Ratio of PF and Surfactant

The mixed solutions of PF and TritonX-100 were designed with volumes of 8:1, 7:1, 6:1, 5:1, 4:1, 3:1, 2:1, 1:1, 1:2, 1:3, 1:4, 1:5, 1:6, and 1:7, which were prepared in advance. After that, the whatman1 filter paper of 10 mm × 10 mm was cleaned three times and then placed in the mixed solution for 2 h. The remaining experimental steps were the same as in Section 2.4.1.

#### 2.4.3. Soaking Time

PF and TritonX-100 were configured as a mixed solution with a volume ratio of 8:1. The 10 mm × 10 mm whatman1 filter paper was washed with deionized water three times and then put into the mixed solution. Whatman1 filter paper was put into the mixed solution and soaked for 1 h, 2 h, 3 h, 4 h, 5 h, 6 h, 7 h, 8 h, and 9 h. The remaining experimental steps were the same as in Section 2.4.1.

### 2.5. Optimizing Experimental Conditions

The pH of the test solution, the color development time for PF-modified test strips, the influence of interfering ions, and the type of masking agent were used as four experimental factors to optimize the experimental conditions of the detection process. The optimal experimental results in experiment Section 2.4 (the type of surfactant is TritonX-100; volume ratio of PF and TritonX-100 is 8:1, and the soaking time is 5 h) are used in experiment Section 2.5.

#### 2.5.1. The pH of the Test Solution

Whatman1 filter paper was put into a mixed solution of PF and TritonX-100 with a volume ratio of 8:1 and then shaken for 5 h to make Mo (VI) test strips. The 0.6 mg L^−1^ Mo (VI) solution was placed in a 25 mL test tube, and then 0.1 mol L^−1^ hydrochloric acid and 0.1 mol L^−1^ sodium hydroxide solution were added drop-wise to adjust the pH value of the Mo (VI) solution to 1, 2, 3, 4, 5, 6, 7, 8, 9, and 10. The subsequent experimental steps were the same as in Section 2.4.1.

#### 2.5.2. The Color Development Time

Whatman1 filter paper was put into a mixed solution of PF and TritonX-100 with a volume ratio of 8:1 and then soaked for 5 h to make Mo (VI) test strips. The 0.6 mg L^−1^ Mo (VI) solution was put into a 25 mL test tube, and the pH was adjusted to 6. After that, the Mo (VI) test strips were put in the Mo (VI) solution and left to stand for 1 min before being taken out. After waiting for 2.5 min, 5 min, 7.5 min, 10 min, 12.5 min, 15 min, 17.5 min, 20 min, 22.5 min, 25 min, 27.5 min, 30 min, 32.5 min, 35 min, and 37.5 min, Mo (VI) test strips were photographed. The subsequent experimental steps were the same as in Section 2.4.1.

#### 2.5.3. The Influence of Interfering Ions

Whatman1 filter paper was put into a mixed solution of PF and Triton X-100 with a volume ratio of 8:1 and then shaken for 5 h to make Mo (VI) test strips. Interfering ions (2.5 mg L^−1^) were added to a 0.8 mg L^−1^ of Mo (VI) solution, namely K^+^, Na^+^, Ca^2+^, Cu^2+^, Zn^2+^, Mn^2+^, Cr^6+^, Fe^3+^, Mg^2+^, C_2_O_4_^2−^, Cl^-^, CO_3_^2−^, NO_3_^2−^, Ni^2+^, Al^3+^, and Cd^2+^ [43], while a 0.6 mg L^−1^ Mo (VI) solution without interfering ions was used as a control. The pH of all solutions to be tested was adjusted to 6. The Mo (VI) test strips were put into the mixed solution for 1 min and then taken out. After waiting for 2.5 min, the Mo (VI) test strips were photographed. An interference was considered negligible when the relative deviation of the signal (S_A_) compared with the control was within ±5%, which was adopted as a practical acceptance criterion for rapid screening and consistent with the agreement threshold used in real-sample comparison with the reference method in this work. If the deviation exceeded ±5%, the interferent concentration was progressively reduced until the deviation fell within ±5%, and the minimum tolerated concentration was recorded.

#### 2.5.4. Type of Masking Agent

Whatman1 filter paper was put into a mixed solution of PF and TritonX-100 with a volume ratio of 8:1 and then shaken for 5 h to make Mo (VI) test strips. Then, 2 mL of 0.02 mol L^−1^ EDTA mixed solution, 2 mL of 100 g L^−1^ ascorbic acid, and 2 mL of 0.02 mol L^−1^ EDTA mixed solution + 2 mL of 100 g L^−1^ ascorbic acid were added to the mixed solution of 2.5 mg L^−1^ Fe^3+^ and 0.8 mg L^−1^ Mo (VI). 0.8 mg L^−1^ Mo (VI) solution was used as the control group. The pH of all solutions to be tested was adjusted to 6. The Mo (VI) test strips were put into the test solution for 1 min and then taken out and waited for 2.5 min before being photographed. The deviation between the experimental results with the masking agent and the standard results was ≤±5%, indicating that the masking agent has the effect of masking the interference of Fe^3+^ on Mo (VI) test strips. If the deviation was greater than ±5%, it means that the masking agent fails to mask Fe^3+^.

### 2.6. Making of Standard Curve and Standard Colorimetric Card

Mo (VI) test strips were prepared according to the above-mentioned optimal experimental results. Mo (VI) solutions with concentrations of 0, 0.02, 0.04, 0.06, 0.08, 0.1, 0.2, 0.4, 0.6, and 0.8 mg L^−1^ were prepared, and the pH of the solution was adjusted to 6. Mo (VI) test strips were placed in different concentrations of Mo (VI) solution and left standing for 1 min, then taken out and kept for 2.5 min before imaging. Origin (2018 version) was used to fit the linear relationship between the Mo (VI) solution and the A-channel value of the Mo (VI) test strips after color development. A naked-eye colorimetric standard color chart was made from photographs taken. An A-value colorimetric card was made with the picture after the L and B channels were turned off in the LAB color space of Photoshop CS 6 in trial edition.

### 2.7. Detection of Urban Water Samples

Surface water (Live Water Park in Chengdu, Sichuan Province) was used to verify the practicality of the Mo (VI) test strips. The actual water samples collected were immediately taken back to the laboratory for vacuum filtration equipment to filter out excess impurities. Then 2 mL of 0.02 mol L^−1^ EDTA was added to the actual water sample to remove the influence of interfering ions. Mo (VI) test strips were used to detect Mo (VI) content in actual water samples according to the steps of Section 2.6. Thiocyanate photometry and a UV-vis spectrophotometer were used as control methods and control instruments.

### 2.8. Stability Experiment of Mo (VI) Test Strips

The Mo (VI) test strips made under the optimal experimental conditions were put in a sealed plastic bag and stored at room temperature. A piece of Mo (VI) test strips was taken out every other day to test the 0.6 mg L^−1^ Mo (VI) solution, and the test results were compared with the standard colorimetric card. Experiments were stopped due to data errors of ≥5% between test results and standard results.

## 3. Results and Discussion

### 3.1. PF-Modified Test Strips as Affected by Type of Surfactant, Volume Ratio of PF and Surfactant, and Soaking Time

**Figure 3:**
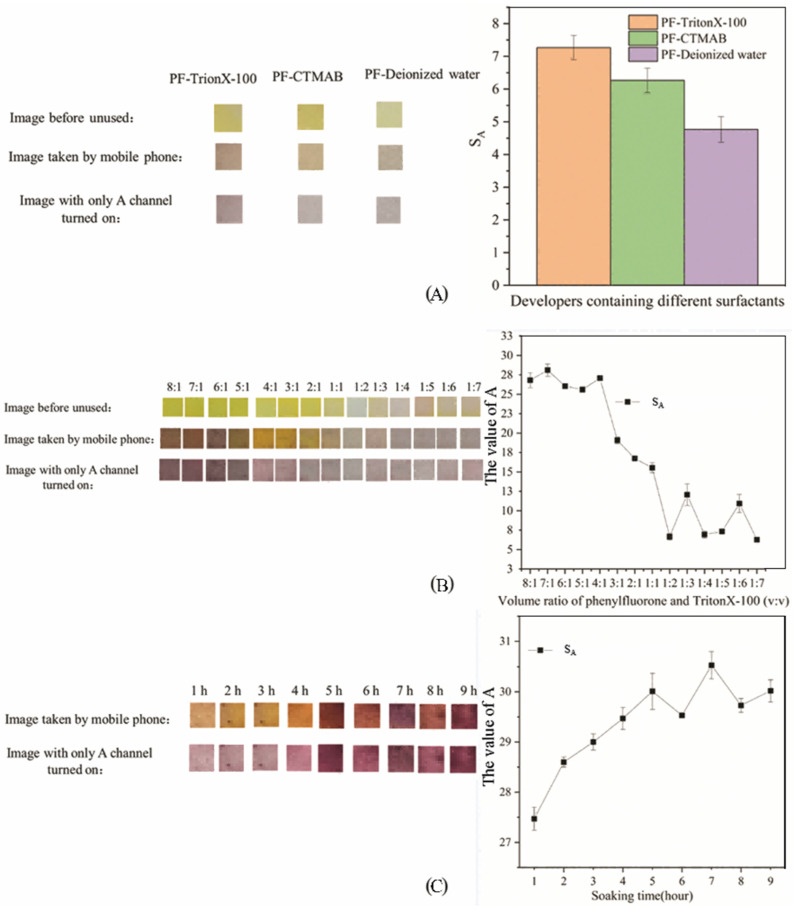
Results of the modification experiment of PF-modified test paper (**A**): Experimental results of different surfactants; (**B**): Experimental results of different volume ratios of PF; (**C**): Experimental results of different soaking times.

The influence of surfactant type, the volume ratio of PF to surfactant, and soaking time on the test results of PF-modified test strips is shown in Figure 3, which is composed of an image before use, an image taken with a mobile phone, an image with only the A channel turned on, and a change curve of the average A-channel value.

As can be seen from Figure 3, the PF-modified test paper with Triton X-100 as the surfactant changes from yellow to orange during the detection process, and appears purple when only the A channel is turned on. It can be seen from the right panel of Figure 3A that S_A_ with Triton X-100 as the surfactant was the highest, followed by CTMAB, and the lowest was PF alone. The experimental results can be analyzed from the chemical structure. PF is an acidic triphenylmethane derivative reagent containing multiple hydroxyl groups or o-hydroxycarbonyl groups, which can form a closed-ring functional group with metal ions [44]. With the solubilization and sensitization effects of surfactants, PF reagents are widely used in the determination of metal ions [45]. The addition of Triton X-100 makes the color development results of PF-modified test strips more obvious, which is reflected in the increased chroma of the image, and the color becomes darker than before by naked-eye observation. Surfactants are generally divided into anionic/cationic surfactants, amphoteric surfactants, nonionic surfactants, compound surfactants, and other surfactants [46]. These surfactants can provide solubilization, sensitization, enrichment effects, and improve anti-interference performance and selectivity in analytical chemistry [47]. CTMAB is a cationic surfactant, which is characterized by nitrogen atoms with lone pairs of electrons in the molecule [48]. Triton X-100 is a nonionic surfactant, which is characterized by ether groups that do not dissociate in aqueous solutions as the main hydrophilic groups [49]. The ether group of nonionic surfactants reacts with the hydroxyl group of PF to form hydrogen bonds, while ionic surfactants are difficult to combine with PF [50]. There is no dissociable group on the 9-position benzene ring in the PF molecule, and the PF molecule is very weak in polarity when the reaction system is neutral [51]. The affinity between Triton X-100 molecules and PF molecules is stronger than that between Triton X-100 molecules, which facilitates the aggregation of PF molecules and produces additional sensitization [52]. Subsequent experiments used Triton X-100 as a surfactant to make PF-modified test strips.

It can be seen from Figure 3B that when the volume ratio was 4:1, the PF-modified test strips were golden yellow, orange yellow, and purple, which was conducive to observing the test results by colorimetry. The average A-channel value at this time was also higher. In subsequent experiments, a 4:1 mixed solution of PF and Triton X-100 was prepared to produce PF-modified test strips. When the volume ratio of PF to Triton X-100 was 4:1, the color result of PF-modified test strips was the most obvious, suggesting that hydrogen-bonding interactions between the ethoxy groups of Triton X-100 and the phenolic hydroxyl groups of PF may be favored at this ratio; however, the specific number of hydroxyl groups involved cannot be confirmed in the present work. When the volume ratio was greater than 4:1, all Triton X-100 molecules could combine with four PF molecules to reach saturation, and the excess PF molecules could directly combine with a small amount of Mo (VI), which may explain why S_A_ remains high when the volume ratio is 8:1 to 4:1. Based on the reported stoichiometry of PF:Mo (VI) = 3:1 in the literature [32], we hypothesize that under the 4:1 (PF:Triton X-100) preparation condition, the overall association among PF, Mo (VI), and Triton X-100 could be approximated as 12:4:3. However, this ratio is proposed as a working hypothesis and should not be considered a confirmed composition, because no direct spectroscopic evidence (e.g., Job’s plot, FTIR, NMR, or MS) was obtained in this study to verify the complex stoichiometry.

It can be seen from Figure 3C that when the soaking time was 4 h, the PF-modified test strips were golden yellow, orange yellow, and purple, which helps colorimetry to distinguish the test results. In subsequent experiments, the soaking time was set to 4 h make PF-modified test strips. Soaking time has a certain effect on S_A_ and the color of PF-modified test strips. This may be because Triton X-100 needs to reach a certain concentration in the solution before it begins to form molecularly ordered aggregates [53]. Triton X-100 ordered aggregates in solution can contact PF molecules to form composite micelles. The micelles are unstable during the formation process and are easily affected by the external environment, causing breakage and reconnection [54]. Therefore, the binding process of PF and Triton X-100 takes a certain amount of time to form a stable complex compound.

### 3.2. Results of Mo (VI) Test Strip Impacted on Main Factors

**Figure 4:**
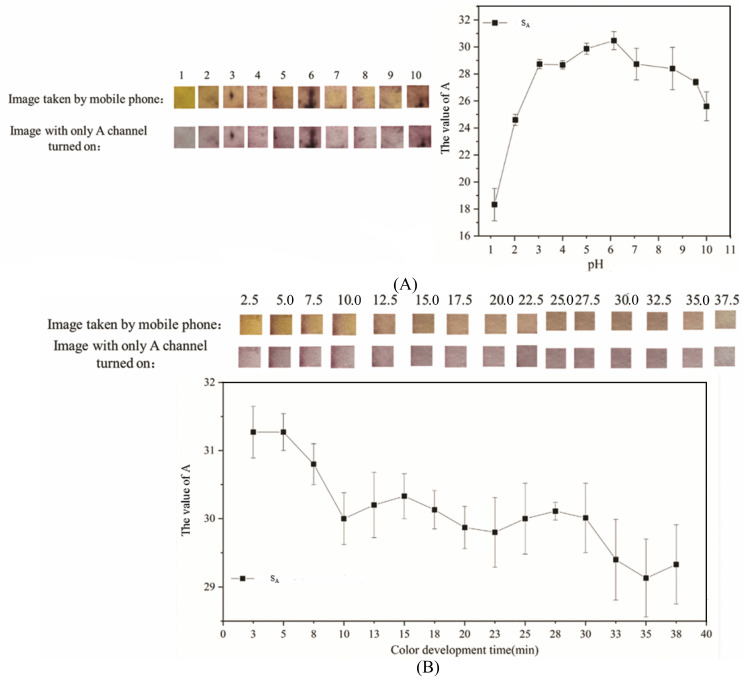
Results of optimizing experimental conditions (**A**): Experimental results of different pH; (**B**): Experimental results of different color development time.

The influence of pH and color-development time on the test results of PF-modified test strips is shown in Figure 4, which is composed of an image before use, an image taken with a mobile phone, an image where only the A channel is turned on in Photoshop, and the change curve of the average A-channel value.

**Figure 5:**
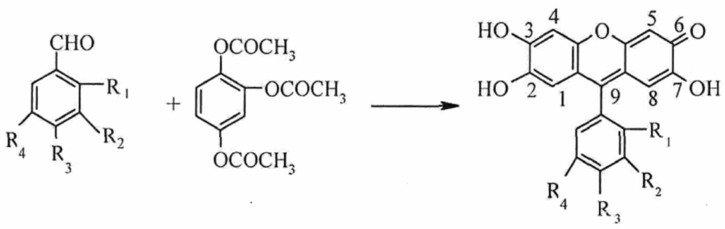
Proposed formation route and chemical framework of PF (phenylfluorone), highlighting the hydroxyl groups (O-donor sites) responsible for Mo (VI) coordination and color development (see Figure 1).

It can be seen from Figure 4A that when the pH was 6, the PF-modified test strips were orange-yellow and purple, respectively, which is conducive to observing the experimental results by colorimetry. S_A_ at this time was also very high. The pH of the Mo (VI) solution was set to 6 in subsequent experiments. In a weakly acidic environment, the color reaction of PF-modified test strips in the detection of Mo (VI) is very obvious, which is the same as the experimental results of Cui et al. [32]. PF contains multiple phenolic hydroxyl groups that act as O-donor coordination sites. As illustrated in Figure 5, these hydroxyl groups originate from the PF framework and provide the binding sites responsible for complexation with Mo (VI) [55]. Combined with the complexation stoichiometry and color-development mechanism shown in Figure 1 (PF:Mo (VI) = 3:1), Figure 5 helps explain the pH-dependent response observed experimentally (Figure 4A): alkaline conditions weaken/alter the reactive colored form of PF and thus reduce complex formation, whereas strongly acidic solutions protonate the hydroxyl groups and introduce H^+^ competition, both leading to a decreased signal [56].

It can be seen from Figure 4B that when the color development time was 2.5 min, the PF-modified test strips were yellow-orange and purple, respectively, which is conducive to observing the experimental results by colorimetry, and S_A_ was also at its highest value. Previous literature indicates that the detection time of Mo (VI) in water by PF is generally within 10–20 min. [55,57]. PF-modified test strips only need to wait 2.5 min to get a stable color result, which illustrates the rapidness of the detection method.

When PF-modified test strips were used to detect Mo (VI) with a concentration of 0.8 mg L^−1^ in water, the maximum allowable concentrations of various anions and cations are shown in Table 1.

It can be seen from Table 1 that the detection of Mo^6+^ in water by PF-modified test strips is more vulnerable to the interference of metal cations, because the oxygen atom in the hydroxyl group of the PF molecule has two lone pair electrons, which are easy to form coordination bonds with the empty orbits of metal ions, thus forming a stable metal complex [44].

**Figure 6:**
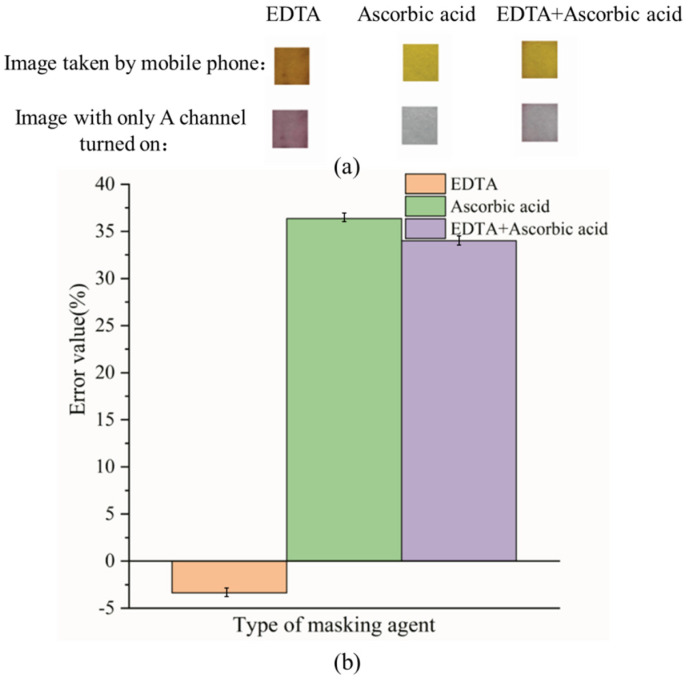
The results of the type of masking agent (**a**): Image results of different types of masking agents; (**b**): S_A_ for different types of masking agents.

The reason for choosing a masking agent based on the ability to mask Fe^3+^ is that the experimental results of interfering ions show that the concentration of Fe^3+^ has a great influence on the experimental results. The experimental results of EDTA, ascorbic acid, and EDTA + ascorbic acid used as masking agents to mask 2.5 mg L^−1^ Fe^3+^ are shown in Figure 6.

Figure 6 shows that with EDTA as a masking agent, the images taken with a mobile phone and the images with only the A channel turned on were orange-red and purple, respectively, which is conducive to observing the experimental results by colorimetry, and the error value at this time is also less than 5%. EDTA and Fe^3+^ also form a complex with a molar ratio of 1:1, wherein the carboxyl group and nitrogen atom on EDTA coordinate with Fe^3+^ [58]. The simultaneous presence of ascorbic acid and EDTA in the solution reduces the masking ability of EDTA, which may be because ascorbic acid reduces Fe^3+^ to Fe^2+^ [59].

### 3.3. Standard Curve and Standard Colorimetric Card

**Figure 7:**
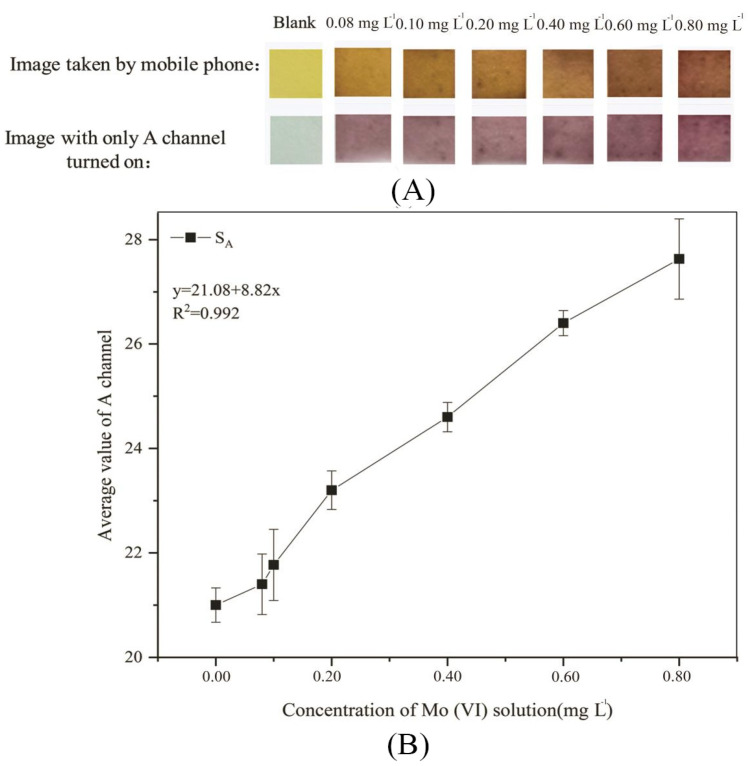
Experimental results of standard colorimetric card and standard curve (**A**): Standard colorimetric card; (**B**): Standard curve.

PF-modified test strips were soaked in a mixed solution of PF and Triton X-100 with a volume ratio of 4:1 for 4 h, and then used to detect Mo (VI) solutions at concentrations of 0.08, 0.1, 0.2, 0.4, 0.6, and 0.8 mg L^−1^ with the pH adjusted to 6. The images of the experimental results were taken with a mobile phone after 2.5 min. The image taken with a mobile phone, the image with only the A channel turned on in Photoshop, and the change curve of the average A-channel value are shown in Figure 7.

As can be seen in Figure 7A, the color change of PF-modified test strips when detecting Mo (VI) solutions with concentrations of 0.08, 0.1, 0.2, 0.4, 0.6, and 0.8 mg L^−1^ can be observed by the naked eye. It can be seen from the images taken with a mobile phone that with the increase in Mo (VI) concentration, PF-modified test strips first change from yellow to orange, and then to orange-red. It can be seen from the image with only the A channel turned on that with increasing Mo (VI) concentration, PF-modified test strips first change from gray to purple, and then to purplish-red. The combination of the image captured with a mobile phone and the image with only the A channel turned on is the standard colorimetric card of PF-modified test strips, which can help roughly determine the concentration range of Mo (VI) in water by distinguishing between the colors with the naked eye. The standard colorimetric card has the advantages of low cost, simple operation, no need for professional instruments, and rapid results, so it is very suitable for rapid detection in the field environment [60]. It can be seen from Figure 7B that the linear relationship between the Mo (VI) concentration (x) and S_A_ (y) meets y = 21.08 + 8.82x (R^2^ = 0.992) when the Mo (VI) concentration is 0–0.8 mg L^−1^.

To highlight the advantages of the proposed PF-modified test strips, we compared them with representative Mo (VI) determination methods (Table 2). Although some instrumental techniques provide lower detection limits, they usually rely on expensive equipment and are less suitable for on-site screening. In contrast, the proposed method enables rapid (2.5 min) and low-cost field screening, with an instrument-free visual detection limit of 0.08 mg L^−1^ and a quantitative range of 0.08–0.8 mg L^−1^ when combined with smartphone/LAB analysis.

### 3.4. Mo (VI) Detection in Real Water Samples

Three points are selected as representative water samples in the Chengdu Living Water Park, namely the sampling point of the inflow (104.096721689 S, 30.676210900 N), the sampling point of the middle water (104.098630609 S, N30.674642491 N), and the sampling point of the effluent (104.099915200 S, N30.673549226 N) .

**Figure 8:**
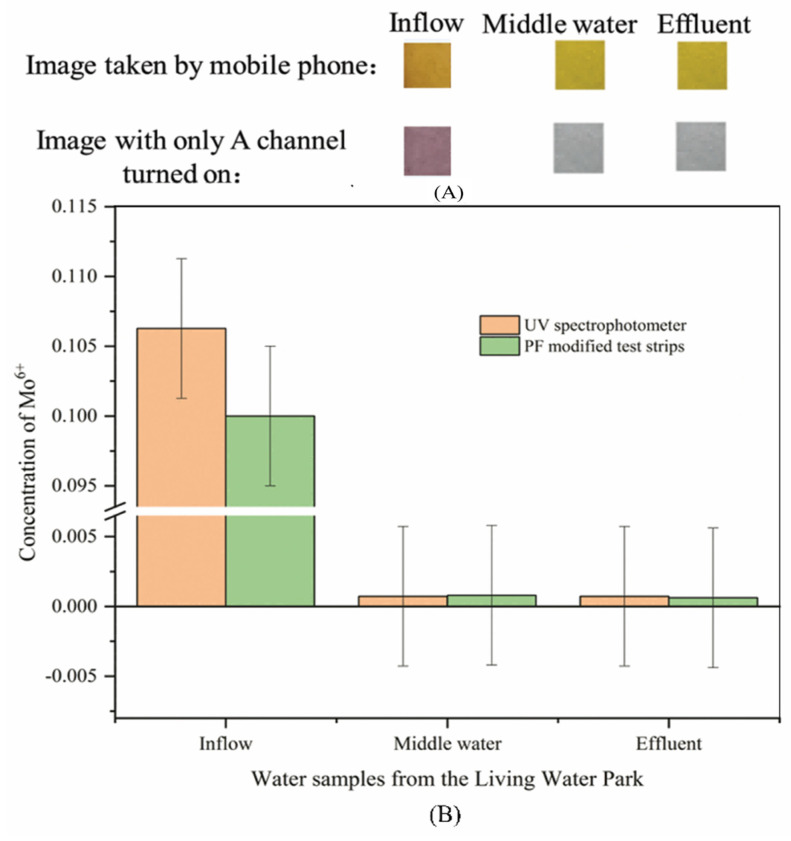
Test results of Mo (VI) in water samples of Living Water Park (**A**): Image results of PF-modified test strips detect real water samples; (**B**): PF-modified test strips and UV spectrophotometry on the Mo (VI) detection results of water samples in the Living Water Park.

The image of a real water sample taken with a mobile phone, the image of a real water sample, with only the A channel turned on in PS, and the change curve of the average value of the A channel are shown in Figure 8.

It can be seen from Figure 8A that when detecting inflow, the PF-modified test strips changed from golden-yellow to orange in the image taken with a mobile phone, and purple in the image with only the A channel turned on. When detecting middle water and effluent, PF-modified test strips change from golden-yellow to yellow in the image taken with a mobile phone, and gray in the image with only the A channel turned on. In Figure 8B, experimental results of UV spectrophotometry and PF-modified test strips show that the content of Mo (VI) in the influent is the highest, and the difference in error between the two results is less than 5%. This indicates that the accuracy of the PF-modified test strip is reasonable. The results of UV spectrophotometry show that the content of Mo (VI) in middle water and effluent was lower than the detection limit of PF-modified test strips by 0.08 mg L^−1^, which was the reason that PF-modified test strips had no obvious color reaction in the detection process. In this case, the value of the A channel used to obtain the image in the LAB color space is then brought into the standard curve to calculate the content of Mo (VI). After the influent of the live water park flows through the anaerobic tank, facultative tank, plant pond bed system, and fish pond, and other water purification systems in turn [65], the content of Mo^6+^ in the middle water and outlet water is significantly lower than or less than 0.07 mg L^−1^, which is the highest content of Mo in surface water specified in the “Environmental quality standard for surface water” (GB 3838-2002) [2]. UV spectrophotometry and PF-modified test strips are less than 5% between different concentrations of experimental results, indicating that PF-modified test strips are scientific and accurate as a field-testing instrument.

### 3.5. Stability Experiment Results of Mo (VI) Test Strips

**Figure 9:**
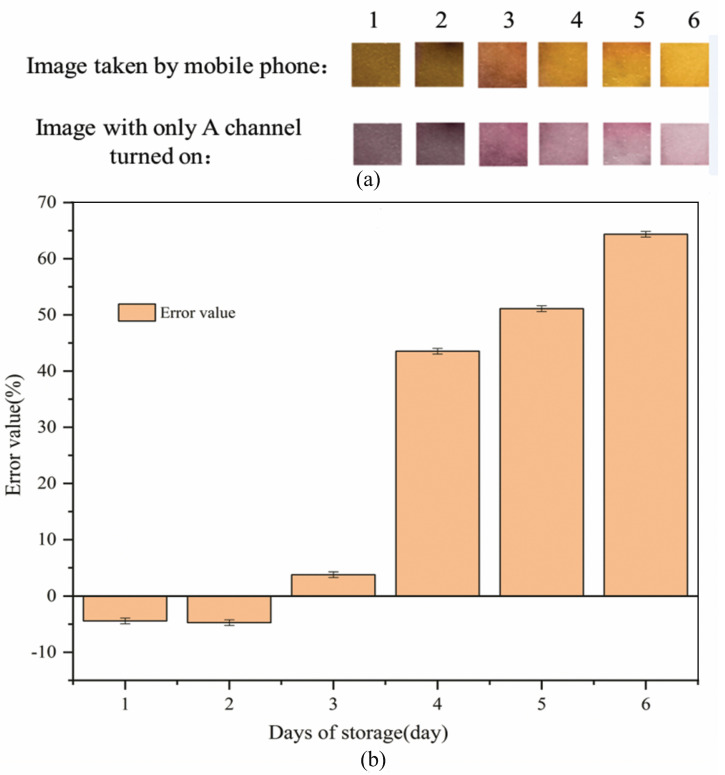
Results of the stability experiment of Mo (VI) test strips (**a**): Image results of the stability experiment of Mo (VI) test strips; (**b**): Average value of A channel for the stability experiment of Mo (VI) test strips.

PF-modified test strips were placed directly in air for a certain amount of water to detect 0.8 mg L^−1^ Mo (VI) in water. The stability results of the PF-modified test strips are shown in Figure 9.

It can be seen from Figure 9a that the PF-modified test strips change from orange-red to orange and finally to yellow in the images taken with a mobile phone as the storage days increase. It can be seen from the image with only the A channel turned on that the PF-modified test strips change from dark purple to purple, and finally to purple-pink. It can be seen from Figure 9b that as the storage days increase, the error value of the experimental results gradually increases. The error value for 1–3 days of storage is less than 5%. The error value for s4–6 days of storage is greater than 5%, which means that the PF-modified test strips need to be used to detect Mo (VI) in water within 3 days after sampling. The reduced stability of PF test strips may be because Triton X-100 undergoes thermal oxidation in air or oxygen-containing gas [66]. Oxygen can significantly increase the evaporation rate of Triton X-100, which evaporates at the rate of ~16 kcal mol^−1^, independent of oxygen concentration [67]. Vacuum plastic sealing is a good way to isolate air and prolong the stability of PF-modified test strips [1].

**Figure 10:**
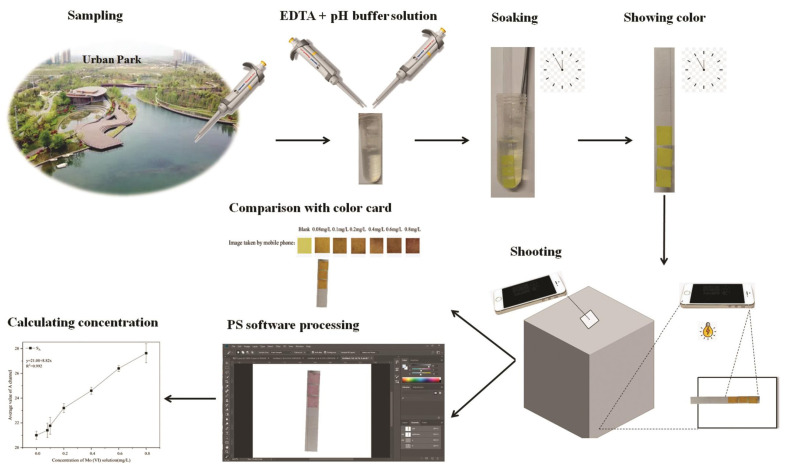
Demonstrations for detection device for Mo (VI) in portable field ambient water samples.

In order to further develop the actual application value of this study, we designed a detection device for Mo (VI) in a portable field ambient water sample, including PF-modified test strips, pH buffer solution, EDTA solution, a light shield with a built-in light source, a standard color card, a mobile phone, and a shooting pad. The detection method is divided into five steps (see Figure 10).

Step 1: Remove interfering ions and regulate the pH. A 25 mL water sample to be tested was added to 2 mL of EDTA solution and mixed thoroughly. A pH buffer solution was used to adjust the pH of the water sample to 6.

Step 2: Detection. PF-modified test strips were soaked for 1 min in the water sample and then removed, with excess solution shaken off.

Step 3: Taking experimental photos. After 2.5 min, PF-modified test strips were placed in the photo area of the shooting pad. After covering with the light shield, the phone was used to take a photo through the small hole in the upper portion of the hood.

Step 4: Comparison with the standard color cards. Experimental photos were used to compare with standard color cards to preliminarily determine the concentration range of Mo (VI) in the water sample. If the Mo (VI) concentration is less than 0.08 mg L^−1^ or between 0.6 and 0.8 mg L^−1^, the exact concentration of Mo (VI) in the water sample to be tested can be obtained by step 5. The Mo (VI) concentration is higher than 0.8 mg L^−1^, which indicates that Mo (VI) exceeds the detection range in the water sample.

Step 5: Photoshop analysis. Experimental photos were imported into the LAB color space in Photoshop on a mobile phone. The A-channel value of the color zone was entered into a standard curve to calculate the Mo (VI) concentration in the water sample.

After the portable Mo (VI) detection device is validated in the laboratory and in the field, it can be promoted on a large scale to demonstrate its economic value and environmental benefits.

## 4. Conclusions

In summary, a new type of PF-modified test strip was designed and used to quickly detect Mo (VI) in urban park water samples. It immediately generated a visible color reaction when PF-modified test strips came into contact with Mo (VI), and the resulting images were used to create a standard colorimetric card for quantitative analysis. The LAB color space was used to quantify the color depth, and the A-channel value was used to construct a standard curve for quantitative analysis. The standard colorimetric card can be used to detect Mo (VI) concentrations of 0.08 to 0.8 mg L^−1^ in water. A blank (0 mg L^−1^) was used as the baseline reference. The calibration curve was established over 0.08–0.8 mg L^−1^ for image-assisted semi-quantitative analysis. The PF-modified test strips provided in this study are easy to prepare, portable, rapid, precise, and low-cost. PF-modified test strips can be more widely used in environmental detection after the problem of instability during storage is solved.

Nevertheless, the present work has some limitations in terms of comprehensive validation. More extensive replicate measurements at multiple concentration levels and across different days are required to fully characterize method precision and operational robustness under field-like conditions. In addition, systematic spike–recovery tests in a broader range of real-water matrices (e.g., varying ionic strength and organic matter) would further strengthen the assessment of accuracy and matrix effects. Future work will therefore focus on expanding validation datasets and improving resistance to matrix/interference effects to support reliable field deployment.

## Figures and Tables

**Table 1:** Results of interference-ion experiments.

Interfering Ions	Maximum Allowable Concentration (mg/L)	Interfering Ions	Maximum Allowable Concentration (mg/L)
K^+^	1.25	Fe^3+^	0.1
Na^+^	1.25	Mg^2+^	2.5
Ca^2+^	2.5	C_2_O_4_^2−^	1.25
Cu^2+^	1.25	Cl^-^	No effect
Zn^2+^	2.5	CO_3_^2−^	No effect
Mn^2+^	1	NO_3−_	No effect
Cr^6+^	2.5	Ni^2+^	1
Cd^2+^	0.25	Al^3+^	0.5

**Table 2:** Comparison of key performance parameters for representative Mo (VI) determination methods.

Method (Principle)	LOD	Linear Range	Response Time	Estimated Cost	Ref.
This work (PF test strip + smartphone/LAB)	0.08 mg L^−1^ (visual)	0.02–0.8 mg L^−1^	2.5 min	Very low	This work
Phenylfluorone on fibrous sorbent (solid-phase color scale)	0.02 mg L^−1^	0.025–0.4 mg L^−1^	10 min	Low	Dedkova et al., 2000 [61]
Polymer-membrane optical sensor (optode; spectrophotometry)	2.5 × 10^−8^ M (~0.0024 mg L^−1^)	8.5 × 10^–8^ – 4.4 × 10^−5^ M (~0.008–4.22 mg L^−1^)	3–5 min	Moderate	Amin et al., 2023 [62]
Fluorescent probe (molecular fluorescence)	1.5 × 10^−8^ M (~0.0014 mg L^−1^)	Up to 2.0 µM (~0.192 mg L^−1^)	—	High	Banerjee et al., 2019 [63]
Adsorptive stripping voltammetry (screen-printed electrode)	0.7 ng mL^−1^ (0.0007 mg L^−1^)	—	—	Moderate–high	Rojas-Romo et al., 2020 [64]

## Data Availability

The data presented in this study are available on request from the corresponding author.
